# Glucose derived carbon nanosphere (CSP) conjugated TTK21, an activator of the histone acetyltransferases CBP/p300, ameliorates amyloid‐beta 1–42 induced deficits in plasticity and associativity in hippocampal CA1 pyramidal neurons

**DOI:** 10.1111/acel.13675

**Published:** 2022-08-12

**Authors:** Akash K. Singh, Sin H. Neo, Christine Liwang, Karen K. L. Pang, Jason C. K. Leng, Sarmistha H. Sinha, Mahesh S. Shetty, Madavan Vasudevan, Vinay J. Rao, Ila Joshi, Muthusamy Eswaramoorthy, Maria V. Pavon, Ang R. Sheila, Sheeja Navakkode, Tapas K. Kundu, Sreedharan Sajikumar

**Affiliations:** ^1^ Transcription and Disease Laboratory, Molecular Biology and Genetics Unit Jawaharlal Nehru Centre for Advanced Scientific Research Bengaluru India; ^2^ Department of Physiology Yong Loo Lin School of Medicine, National University of Singapore Singapore Singapore; ^3^ Lee Kong Chian School of Medicine, Nanyang Technological University Singapore Singapore; ^4^ Department of Neuroscience and Pharmacology Iowa Neuroscience Institute, Carver College of Medicine, University of Iowa Iowa City Iowa USA; ^5^ Theomics International Pvt Ltd Bangalore India; ^6^ Chemistry and Physics of Materials Unit Jawaharlal Nehru Centre for Advanced Scientific Research Bengaluru India; ^7^ Division of Neuroscience and Aging Biology CSIR‐Central Drug Research Institute Sector 10 Lucknow Uttar Pradesh India; ^8^ Life Science Institute Neurobiology Programme (LSI) National University of Singapore Singapore Singapore; ^9^ Healthy Longevity Translational Research Programme Yong Loo Lin School of Medicine, National University of Singapore Singapore Singapore

**Keywords:** CREB‐binding protein, long‐term potentiation, synaptic tagging, synaptic tagging/ capture, TTK21

## Abstract

The master epigenetic regulator lysine acetyltransferase (KAT) p300/CBP plays a pivotal role in neuroplasticity and cognitive functions. Recent evidence has shown that in several neurodegenerative diseases, including Alzheimer's disease (AD), the expression level and function of p300/CBP are severely compromised, leading to altered gene expression causing pathological conditions. Here, we show that p300/CBP activation by a small‐molecule TTK21, conjugated to carbon nanosphere (CSP) ameliorates Aβ‐impaired long‐term potentiation (LTP) induced by high‐frequency stimulation, theta burst stimulation, and synaptic tagging/capture (STC). This functional rescue was correlated with CSP‐TTK21‐induced changes in transcription and translation. Mechanistically, we observed that the expression of a large number of synaptic plasticity‐ and memory‐related genes was rescued, presumably by the restoration of p300/CBP mediated acetylation. Collectively, these results suggest that small‐molecule activators of p300/CBP could be a potential therapeutic molecule for neurodegenerative diseases like AD.

## Graphical abstract

Exogenous Aß(1–42) oligomers impair high‐frequency stimulation‐ and dopaminergic agonist‐induced synaptic plasticity and associative plasticity in the rat hippocampal area CA1. CSP‐TTK21, a small‐molecule activator of lysine acetyltransferase CBP/p300, rescues such Aß‐impaired synaptic plasticity. CSP‐TTK21 changes the transcriptome of area CA1 of Aß‐treated rat hippocampal slices and rescues the expression of key plasticity‐related proteins (PRPs).

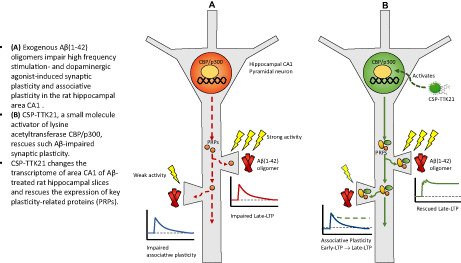

## 1 INTRODUCTION

Alzheimer's disease (AD), the most common neurodegenerative disorder, results in progressive dementia and deterioration of cognitive functioning. Although AD is most recognized by the cellular pathological hallmarks of tau neurofibrillary tangles and Aβ plaques, it is thought that AD‐associated synaptic pathology precedes these histological markers (Selkoe, 2002). Notably, it has been suggested that synaptic dysfunction in the hippocampus, a brain structure important for memory formation, underlies early amnestic symptoms in AD.

Synaptic dysfunctions can be caused by soluble Aβ even before the formation of insoluble plaques and synapse loss through multiple mechanisms. Functionally, soluble Aβ oligomers impair synaptic plasticity—including long‐term potentiation (LTP), a cellular correlate of long‐term memory (Chen et al., 2002; Li et al., 2011), and late‐associative plasticity, as experimentally epitomized by Synaptic Tagging and Capture (STC). The STC model explains how input‐specific, weakly activated synapses can express long‐term synaptic plasticity by capturing plasticity‐related proteins from strongly activated, nearby independent synaptic inputs within a specific time frame (Bin Ibrahim et al., 2022; Frey & Morris, 1997). At the molecular level, Aβ disrupts multiple pathways that are linked to synaptic plasticity, learning, and memory, for example, the MAPK/ERK signaling pathways (Balleza‐Tapia & Pena, 2009; Li et al., 2011). Particularly, Aβ interferes with the activity of cyclic adenosine monophosphate (cAMP) response element‐binding protein (CREB) (Vitolo et al., 2002), a regulator molecule critical for the expression of genes related to synaptic plasticity and memory (Kandel, 2012).

In addition to Aβ pathology, it is increasingly appreciated that AD is also associated with epigenetic aberrations (Klein et al., 2019; Marzi et al., 2018; Nativio et al., 2018; Singh et al., 2018). In an AD‐related mouse model of neurodegeneration, histone acetylation was found to be significantly down‐regulated in the promoter regions of neuronal plasticity‐related genes, hence impeding their expression (Graff et al., 2012). Furthermore, in both AD mouse model and human patient samples, the reduced expression of synapse‐ and learning‐related genes was concomitant with a decrease in epigenomic marker levels in their enhancer and promoter regions (Gjoneska et al., 2015). Specifically, these enhancer regions with decreased acetylation were largely bound by CREB‐binding protein (CBP), a transcriptional coactivator important for histone acetylation, synaptic plasticity, as well as learning and memory (Barrett et al., 2011). Hence, reinstating the epigenome and transcriptome presents an enticing therapeutic strategy in restoring neuroplasticity and memory in AD conditions.

TTK21, an activator of the acetyltransferase function of CBP/p300 when conjugated with glucose‐based carbon nanospheres (CSP), hence termed as CSP‐TTK21 (Chatterjee et al., 2013), restored acetylation of histone H2B in the hippocampus of THY‐Tau22 mice, a mouse model with AD‐related Tau pathology (Chatterjee et al., 2018). Moreover, CSP‐TTK21 also up‐regulated the expression of plasticity‐related genes and improved spatial memory in these mice.

In this study, we found that CSP‐TTK21 could also rescue synaptic plasticity deficits caused by acute Aβ exposure in the CA1 area of rat hippocampal slices. We showed that CSP‐TTK21 could restore long‐term potentiation and late‐associative plasticity induced by high‐frequency tetanization and theta burst stimulation. Furthermore, we demonstrated that this rescue of functional synaptic plasticity was concomitant with the CSP‐TTK21‐mediated restoration of plasticity‐related gene expression.

## 2 MATERIALS AND METHODS

### 2.1 Hippocampal slice preparation and electrophysiology

A total of 132 hippocampal slices prepared from 66 adult male Wistar rats (5–7 weeks old) were used for electrophysiological recordings. Animals were housed under 12‐h light/12‐h dark conditions with food and water available ad libitum. All experimental procedures using animals were performed in accordance with the protocols approved by the Institutional Animal Care and Use Committee (IACUC) of the National University of Singapore. Briefly, the rats were decapitated after anesthetization using CO_2_. The brains were quickly removed and cooled in 4°C artificial cerebrospinal fluid (ACSF) that contained the following (in mM): 124 NaCl, 3.7 KCl, 1.0 MgSO_4_ .7H_2_O, 2.5 CaCl_2_, 1.2 KH_2_PO_4_, 24.6 NaHCO_3,_ and 10 D‐glucose, equilibrated with 95% O_2_–5% CO_2_ (carbogen; total consumption 16 L/h). Transverse hippocampal slices (400 μm) were prepared from the right hippocampus using a manual tissue chopper. The slices were incubated at 32°C in an interface chamber (Scientific System Design) with an ACSF flow rate of 1 ml/min and a constant supply of carbogen.

**FIGURE 1:**
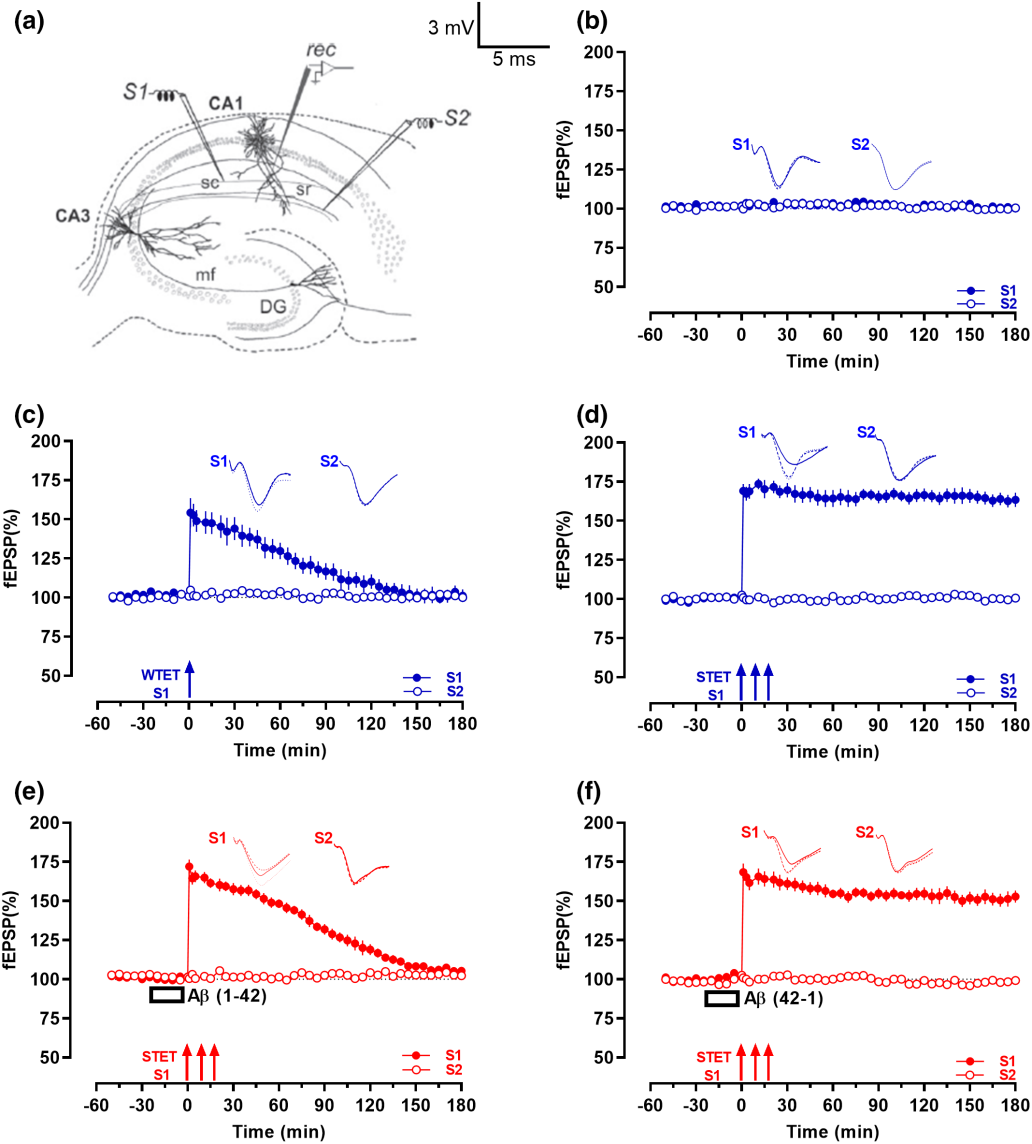
Aβ(1–42) oligomers impaired high‐frequency stimulation‐induced late‐LTP. (a) Schematic depicts location of the recording electrode (rec) and the stimulating electrodes (S1) and (S2) in the area CA1 of hippocampal slices in a two‐pathway experiment. (b) Basal stimulation of the Schaffer collaterals (sc) resulted in stable field excitatory postsynaptic potentials (fEPSP) recording for 3 h (*n* = 7). (c) Delivery of a weak tetanization (WTET; single train of 21 pulses, 100 Hz, 0.2 ms/polarity; broken arrow) led to an early‐long‐term potentiation (early‐LTP)—fEPSP showed an immediate increase but returned to baseline by 95 min (*n* = 7). (d) Delivery of a strong tetanization (STET; 3 trains of 100 pulses,100 Hz, intertrain interval of 10 min, 0.2 ms/polarity; solid arrows) induced a late‐LTP that lasted 3 h (*n* = 7). (e) Bath application of Aβ(1–42) oligomers (200 nM; open box) 20 min before the delivery of STET led to a potentiation of fEPSP that waned over 170 min (*n* = 7). (f) Bath application of the reverse Aβ(42–1) (150 nM, 20 min; open box) did not affect induction of late‐LTP by STET (*n* = 7). All data represent mean ± SEM. Representative fEPSP traces shown in each case recorded at −30 min (solid line); 60 min (dotted line); and 180 min (hatched line). Single arrow represents the time point of application of WTET for the induction of early‐LTP. Triplet of arrows represent the point of application of STET for the induction of late‐LTP. Scale bars for all the traces, vertical: 3 mV; horizontal: 5 ms. sc, Schaffer collaterals; sr, stratum radiatum; mf, mossy fibers; DG, dentate gyrus.

In the electrophysiological recordings, two‐pathway experiments were performed. Two monopolar lacquer‐coated stainless‐steel electrodes (5 MΩ; AM Systems) were positioned within the stratum radiatum of the CA1 region for stimulating two independent synaptic inputs, S1 and S2, of one neuronal population (Figure 1a), thus evoking field excitatory postsynaptic potentials (fEPSP) from Schaffer collateral/commissural‐CA1 synapses. Pathway specificity was tested using the method described in Sajikumar & Korte (2011). An electrode was placed in the CA1 apical dendritic layer for recording the fEPSP. The signals were amplified by a differential amplifier (Model 1700; AM Systems), digitized using a CED 1401 analog‐to‐digital converter (Cambridge Electronic Design), and monitored online.

After 2–3 h incubation, a synaptic input–output curve (afferent stimulation vs. fEPSP slope) was generated. Test stimulation intensity was adjusted to elicit a fEPSP slope of 40% of the maximal slope response for both synaptic inputs S1 and S2. Test stimulation comprising four 0.2‐Hz biphasic constant‐current pulses (0.1 ms per polarity) was used at each time point. In all experiments, a stable baseline was recorded for at least 30 min before plasticity induction. To induce late‐LTP, a “strong” tetanization (STET) protocol, consisting of three high‐frequency stimulations of 100 pulses at 100 Hz (single burst, stimulus duration of 0.2 ms per polarity) with an intertrain interval of 10 min, was used. To induce early‐LTP, a “weak” tetanization (WTET) protocol consisting of a single stimulus train of 21 pulses at 100 Hz (stimulus duration of 0.2 ms per polarity) was used. Theta burst stimulation‐LTP (TBS‐LTP) was induced in hippocampal slices using a protocol that consisted of 50 bursts (consisting of 4 stimuli) at an inter‐stimulus interval of 10 ms. The 50 bursts were applied over a period of 20 s at 5 Hz (Sajikumar & Korte, 2011).

### 2.2 Pharmacology

In vitro oligomer preparation of Aβ(1–42) peptide (AnaSpec) and Aβ(42–1) peptide (Sigma‐Aldrich) was carried out 24 h before the start of electrophysiology experiments using the protocol mentioned in our earlier reports (Baby et al., 2020; Krishna et al., 2016; Sharma et al., 2017). The protein synthesis inhibitors, emetine dihydrochloride hydrate (emetine; Sigma‐Aldrich) and anisomycin (Tocris Biosciences), were stored as concentrated stock solutions of 20 mM in water and 25 mM in DMSO, respectively (Sajikumar et al., 2007). The final concentrations used for Aβ(1–42), Aβ (42–1), emetine, and anisomycin were 200 nM, 150 nM, 20 μM, and 25 μM, respectively. For stocks prepared in DMSO, the final DMSO concentration was kept below 0.1%, a concentration shown to not affect basal synaptic responses (Navakkode et al., 2004). CSP‐TTK21 and CSP were prepared as stock solutions at a concentration of 1.88 mg/ml in deionized water with sonication and kept at −20°C. Prior to application, a working solution of 0.36 μg/ml in ACSF was prepared.

### 2.3 qRT‐PCR

Rat hippocampal slices from 4 biological samples were used for each group (4 slices from each biological samples). Six groups (untreated control, SKF, CSP‐TTK21, SKF+Aβ(1–42), SKF+Aβ(1–42)+CSP, and SKF+Aβ(1–42)+CSP‐TTK21) were collected after incubation and drug application in the electrophysiology set‐up, snap frozen in liquid nitrogen and stored at −80°C. The CA1 region was then microdissected from each slice. Total RNA was isolated from hippocampal slices using Trizol method (Invitrogen Trizol 15,596–026). The slices were homogenized in 200 μl of Trizol reagent, frozen in liquid‐N_2,_ and stored at −80°C. During RNA isolation, samples were thawed on ice followed by centrifugation at 13,000 rpm. The supernatant was subjected to phenol:chloroform extraction. RNA was precipitated using iso‐propanol and RNAse‐free glycogen at RT (15 min). The obtained pellet was washed once in 75% ethanol, air‐dried (5–10 min) and resuspended in DEPC‐treated/RNAse‐free water. Isolated RNA samples were subjected to DNAse treatment (30 min at 37°C) followed by re‐precipitation and resuspension (55°C for 10 min). One μg of RNA was used for cDNA synthesis using MMLV‐reverse transcriptase and oligo‐dT (Sigma: M 1302 and O4387, respectively) as per the manufacturer's instructions. A Step One Plus Real‐Time PCR Detection System (Applied Biosystems; Aihara et al. (2007)) was used to perform qPCR using 2× Takara TB Green Mastermix (ABI), and the respective specific primers are shown in Table 1. The data were analyzed using Step One Software version 2.3 (Applied Biosystems; Aihara et al. (2007)). Fold changes were calculated using the formula: $2^{\left(-\left[C_{t}\;\text{Test}‐C_{t}\;\text{Control}\right]\right)}$. β‐actin was considered as housekeeping gene wherever applicable.

**TABLE 1:** Primer sequences for RT‐PCR analysis

Genes	Primer	Sequences
*IL6*	Forward primer	TCCTACCCCAACTTCCAATGCTC
	Reverse primer	TTGGATGGTCTTGGTCCTTAGCC
*βactin*	Forward primer	AAGTCCCTCACCCTCCCAAAAG
	Reverse primer	AAGCAATGCTGTCACCTTCCC
*Adrb2*	Forward primer	TCGAGCGACTACAAACCGTC
	Reverse primer	GAAGTCCAGAACTCGCACCA
*Wnt10b*	Forward primer	CGTTCACGAGTGTCAGCACC
	Reverse primer	GAACGCACTCTCACGGAAAC
*Tgfb*	Forward primer	CGTCAGACATTCGGGAAGCA
	Reverse primer	GTATCAGTGGGGGTCAGCAG
*Atf5*	Forward primer	TGGGCTGGCTCGTAGACT
	Reverse primer	CCGCTCGGTCATCCAATCA
*S100B*	Forward primer	GGTGACAAGCACAAGCTGAA
	Reverse primer	TGGAGACGAAGGCCATAAAC
*Cxcl1*	Forward primer	GCGGAGAGATGAGAGTCTGG
	Reverse primer	GGCATTGTGCCCTACAAACT
*App*	Forward primer	TTGCATTTTTGAGCTGTTGC
	Reverse primer	CAATGTTGACGAAGGTGTGG

### 2.4 Analysis of mRNA expression by RNA‐Seq

Rat hippocampal slices (2 slices from 2 biological samples were used for each repeat for each group, *n* = 2) from six groups (UT, SKF, CSP‐TTK21, SKF+ Aβ(1–42), SKF+ Aβ(1–42)+CSP, and SKF+ Aβ(1–42)+CSP‐TTK21) were collected after electrophysiology recordings, snap frozen in liquid nitrogen, and stored at −80°C. The CA1 region was then microdissected from each slice. RNA integrity was checked by Agilent Bioanalyzer 2100; only samples with clean rRNA peaks were used. Libraries for RNA‐seq were prepared according to KAPA Stranded RNA‐Seq Kit with RiboErase (KAPA Biosystems) system. Final library quality and quantity were analyzed by Agilent Bioanalyzer 2100 and Life Technologies Qubit3.0 Fluorometer, respectively. 150 bp paired‐end sequencing was performed on Illumina HiSeq 4000 (Illumnia Inc.). Rattus Novergicus genome (Rnor_6.0) was downloaded from GENCODE and indexed using Bowtie2‐build with default parameters. Adapter removal was done using Trim Galore (v 0.4.4), and each of the raw Fastq files was passed through a quality check using FastQC. PCR duplicates were removed using the Samtools 1.3.1 with the help of “rmdup” option. Each of the raw files was then aligned to mm 10 genome assembly using TopHat2 with default parameters for paired‐end sequencing as described in Kim et al. (2013). After aligning, quantification of transcripts was performed using Cufflinks, and then, Cuffmerge was used to create merged transcriptome annotation. Finally, differentially expressed (DE) genes were identified using Cuffdiff. The threshold for DE genes was log2 (fold change) >1.5 for up‐regulated genes and log2 (fold change) <1.5 for down‐regulated genes with *p* < 0.05.

### 2.5 GO enrichment analysis

Gene ontology (GO) analysis was performed in PANTHER (Mi et al., 2013). Significant enrichment test was performed with the set of differentially expressed genes in PANTHER, and Bonferroni correction method was applied to get the best result of significantly enriched biological processes.

### 2.6 Fisher's exact test

Fisher's exact test was performed in PANTHER gene ontology (GO) where *p*‐value significance was calculated based on the ratio of obtained number of genes to the expected number of genes (O/E) considering the total number of genes for the respective pathway in Mus musculus with a FDR of <0.05.

### 2.7 Heatmap and clustering of genes

Unsupervised hierarchical clustering method was performed using Cluster 3.0 (Eisen et al., 1998) with Pearson correlation and average linkage rule. Gene expression data (FPKM of all samples) were taken and log2 transformed. Low expressed (FPKM < 0.05) and invariant genes were removed. Then, genes were centered and clustering was performed based on differential expression pattern of genes and fold change. Finally, the heatmap was visualized in Java TreeView 3.0.

### 2.8 Biological analysis of differentially expressed transcripts and pathway regulatory network modeling

Statistically significant differentially expressed transcripts were subjected to GO and pathway enrichment using DAVID tool. Only those GO and pathways with a FDR score of <=0.05 were considered for further downstream analysis. Key biologically dysregulated GO and pathways along with the differentially expressed genes was provided as an input to Pathreg algorithm from Theomics International Pvt Ltd, for gene regulatory network modeling. The result (nodes and edges) of the Pathreg algorithm was provided as an input to Cytoscape v2.8.2 to identify key nodes and edges that could be representative of the gene regulatory changes upon treatment. Identification of genes involved in AD was obtained from NCBI Gene database, and protein–protein interaction data were obtained from STRING Database manual curation of candidate genes. These differentially expressed genes were then provided as an input to VENNY2.0 (Khan & Mathelier, 2017) for plotting vein diagram to illustrate the rescue of the transcriptional defects by CSP‐TTK21.

### 2.9 Statistical analysis

The time‐matched, normalized data were averaged across replicate experiments and expressed as mean ± SEM. The average percentage values of fEPSP slope per time point were subjected to statistical analysis with GraphPad Prism 6.0. Whenever the data did not conform to Gaussian distribution, nonparametric tests were used. Wilcoxon matched‐pairs signed rank test was used when comparisons were made within group, while Mann–Whitney *U* test was used for comparisons made across two independent samples. In experimental groups where electrophysiological recordings started at −30 min, normalized fEPSP percentage values at specified time points were compared with that of the baseline recorded at the ‐15th min; in cases where recordings started at −50 min, normalized fEPSP at mentioned time points were compared to that of the baseline at the −35th min; similarly, when recordings started at −60 min, normalized fEPSP at specified points were compared with that at the −45th min. Statistical significance was assumed at *p* < 0.05.

For identification of differentially expressed mRNAs test *p*‐value (Unpaired Student's *t* test with Benjamini–Hochberg FDR correction) threshold adjusted for false discovery rate of <0.05 was considered. For Gene Ontology and Pathway Analysis, a *p* < 0.05 (Fischer's exact test) along with FDR correction was considered.

For qRT‐PCR analysis, two‐way anova with Fisher's exact test was performed and statistical significance was assumed at *p* < 0.05.

## 3 RESULTS

### 3.1 Aβ(1–42) oligomers impair late‐LTP

In order to model an AD condition in hippocampal slices in vitro, we first replicated previous findings that bath application of Aβ (1–42) oligomers (200 nM) prior to late‐LTP induction impairs its maintenance (Chen et al., 2002; Krishna et al., 2016; Sharma et al., 2017). We conducted experiments using a two‐pathway experimental design (Figure 1a). Without additional tetanic stimulation, baseline fEPSPs recorded from the two independent synaptic inputs S1 and S2 from CA1 apical dendrites were stable for 3 h (Figure 1b, *n* = 7). The fEPSP values at any recorded time point did not show statistically significant potentiation when compared with its baseline (Wilcoxon test) and between S1 and S2 (*U* test) (all *p‐*values were *p* > 0.05, Wilcoxon test and *p* > 0.05, *U* test). Induction of early‐LTP by Weak Tetanization ((WTET), 100 Hz, 21 pulses, see methods) (Figure 1c, *n* = 7) resulted in an immediate increase in fEPSP that waned over the course of the experiment; the potentiation was statistically significant up to 95 min (*p* = 0.047, Wilcoxon test; *p* = 0.019, *U* test), while the induction of late‐LTP by Strong Tetanizaton ((STET), 3 × 100 Hz, 100 pulses, see methods) (Figure 1d, *n* = 7) resulted in an immediate increase in fEPSP that remained statistically significant at 180 min (*p* = 0.016, Wilcoxon test; *p* = 0.001, *U* test). In contrast to these control conditions, when exogenous Aβ(1–42) oligomer (200 nM) was bath applied 20 min before late‐LTP induction similar to our earlier report STET led to a potentiation that waned over time (Krishna et al., 2016) (Figure 1e, *n* = 7). This potentiation was only statistically significant up to 170 min when compared to its own baseline (*p* = 0.016, Wilcoxon test) and up to 140 min compared to S2 (*p* = 0.005, *U* test). Conversely, no LTP impairment was observed when late‐LTP was induced by STET after the bath application of the reverse Aβ (42–1) oligomers (200 nM, 20 min; Figure 1f, *n* = 7). The potentiation remained statistically significant till the end of the recording period (*p* = 0.016, Wilcoxon test; *p* = 0.001, *U* test). This potentiation was similar in magnitude to the late‐LTP induced in control slices (Figure 1d,f: (1 min; *p* => 0.999); (60 min; *p* = 0.165); (120 min; *p* = 0.097); (180 min; *p* = 0.128) *U* test). In all cases, the baseline potentials remained stable throughout the entire recording period (Wilcoxon test, *p* > 0.05, open circles, Figure 1c‐f).

These control experiments are an important pre‐requisite for investigating synaptic associative experiments such as STC, as it relies on the long‐term functional interactions of strongly and weakly tetanized synaptic inputs within a specific time interval (Bin Ibrahim, Benoy, & Sajikumar, 2022).

### 3.2 CSP‐TTK21 rescues late‐LTP impairment induced by Aβ(1–42) oligomers

**FIGURE 2:**
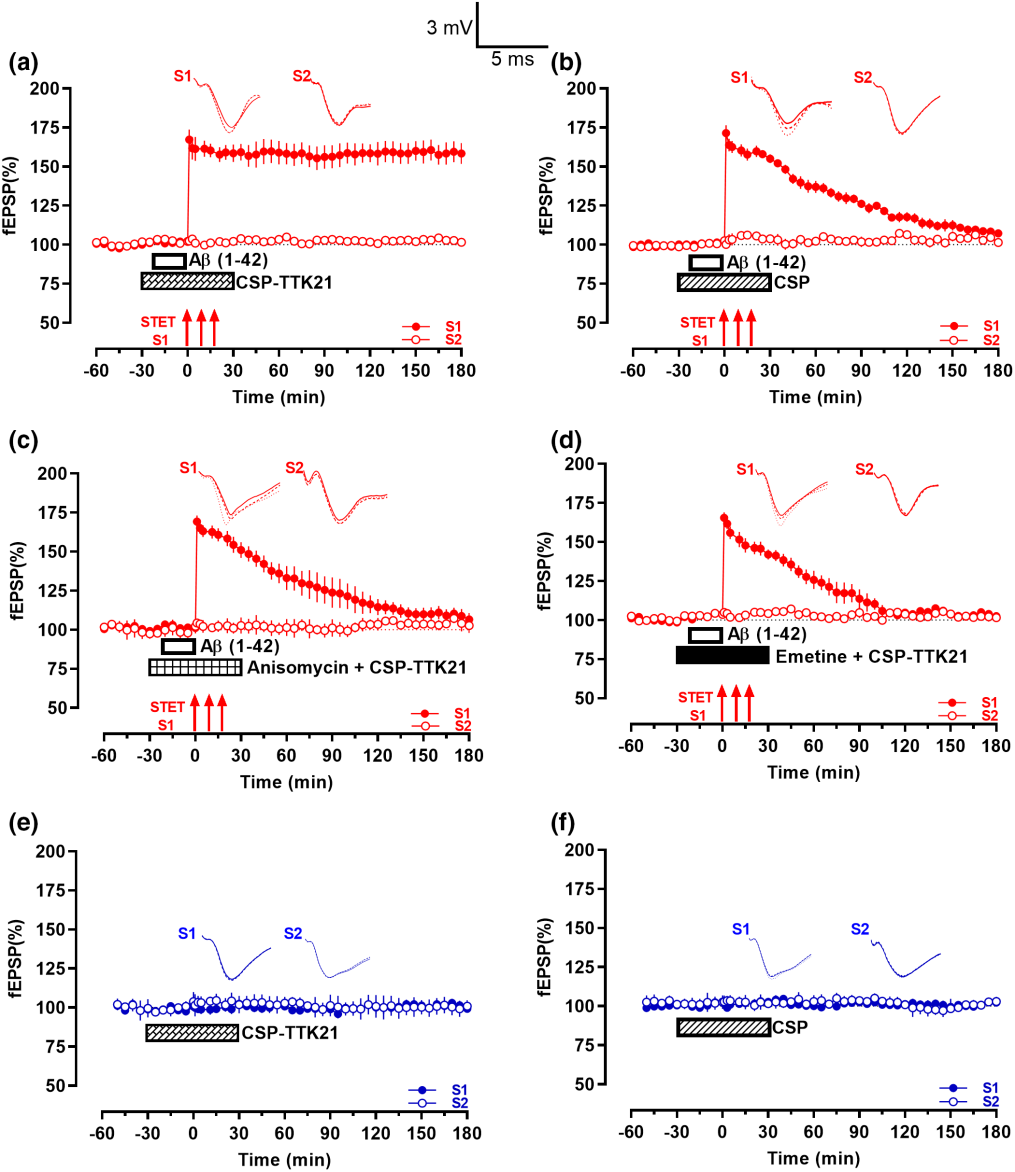
CSP‐TTK21 rescued Aβ(1–42) oligomers‐induced LTP deficits. (a) The CBP/p300 activator CSP‐TTK21 (0.36 μg/ml; crisscross box) was bath‐applied to hippocampal slices 30 min before and 30 min after the first STET tetanus train. In between, Aβ(1–42) oligomers (200 nM; open box) were co‐applied 20 min before STET. In the presence of CSP‐TTK21, STET (solid arrows) led to a late‐LTP that persisted for 3 h (*n* = 7). (b) Similar to that in (a) except CSP (0.36 μg/ml; striped box) was applied instead of CSP‐TTK21. STET led to an early‐LTP that returned to baseline within 140 min (*n* = 6). (c) Similar to that in (a) except that CSP‐TTK21 and the protein synthesis inhibitor Anisomycin (25 μM) were co‐applied for 1 h (checkered box). STET resulted in a potentiation that returned to baseline in 75 min (*n* = 7). (d) Similar to that in (c) except another protein synthesis inhibitor, emetine (20 μM; filled box) was used. STET also led to a potentiation that waned over 55 min (*n* = 6). (e) The bath application of CSP‐TTK21 (0.36 μg/ml; crisscross box) alone for 1 h had no observable effects on basal fEPSP (*n* = 7). (f) Basal fEPSP was not affected by the 1 h bath application of CSP (0.36 μg/ml; striped box; *n* = 6).

We were intrigued to know whether CSP‐TTK21 can rescue Aβ(1–42)‐induced late‐LTP impairment by bath applying CSP‐TTK21 (0.36 μg/ml) for 30 min before and 30 min after LTP induction (Figure 2a, *n* = 7). Even after Aβ(1–42) bath application, late‐LTP induction by STET in the presence of CSP‐TTK21 resulted in a robust potentiation that lasted until the end of the recording period of 180 min (*p* = 0.016, Wilcoxon test; *p* = 0.001, *U* test). Application of CSP alone did not result in any rescue effects (Figure 2b, *n* = 6). fEPSP increased immediately after STET, but decayed to baseline by 140 min when compared to its own baseline (*p* = 0.031, Wilcoxon test) and 125 min compared to S2 (*p* = 0.014, *U* test).

Next, we tested the protein synthesis dependency of CSP‐TTK21‐rescued late‐LTP, because it is a critical pre‐requisite to investigate associative plasticity mechanisms such as STC (Bin Ibrahim, Benoy, & Sajikumar, 2022). Protein synthesis inhibitors, anisomycin (25 μM) or emetine (20 μM), were co‐applied with CSP‐TTK21, respectively (Figure 2c,d). Both inhibitors prevented the long‐term maintenance of LTP even in the presence of CSP‐TTK21, and STET only led to an early‐LTP‐like potentiation. In Figure 2(c), statistically significant potentiation was observed till 75 (*p* = 0.047, Wilcoxon test; *p* = 0.026, *U* test, *n* = 7), while in Figure 2(d), statistically significant potentiation was maintained up to 55 min, when compared to its baseline (*p* = 0.156, Wilcoxon test) and 70 min compared to S2 (*p* = 0.009, *U* test, *n* = 6). Therefore, the above findings show that CSP‐TTK21‐rescued late‐LTP is protein synthesis dependent.

To examine whether application of CSP‐TTK21 or CSP may induce slow‐onset potentiation by themselves, we applied CSP‐TTK21 or CSP alone for 60 min after obtaining a stable baseline (Figure 2e,f). Both CSP‐TTK21 and CSP did not induced any potentiation in both inputs S1 and S2 (*p* > 0.05, Wilcox test; *p* > 0.05, *U* test, Figure 2(e) (*n* = 7) and 2F (*n* = 6)). In addition, baseline control potentials recorded from Figure 2(a–d) reminded stable throughout the entire recording period S2, (Wilcoxon test, *p* > 0.05, open circles).

Next, we were intrigued to know whether the rescue effect of CSP‐TTK21 is present in a more physiological LTP. To test this, we used theta burst stimulation (TBS, see methods) to induce late‐LTP. Induction of TBS late‐LTP resulted in long‐lasting LTP that lasted till 180 min (*p* = 0.016, Wilcoxon test; *p* = 0.001, *U* test) (Figure S1a). In contrast, application of Aβ(1–42) oligomers (200 nM, 20 min) prior to TBS application resulted in a decaying form of LTP (Figure S1b). Statistically significant potentiation was observed only up to 155 min, when compared to its baseline (*p* = 0.016, Wilcoxon) and 125 min when compared to S2 (*p* = 0.012, *U* test). Interestingly, CSP‐TTK21 application 30 min before and after TBS induction rescued Aβ impaired TBS‐late‐LTP, showing stable LTP lasting 180 min (Figure S1c, *p* = 0.016, Wilcoxon test, *p* = 0.001, *U* test). However, application of CSP did not result in any rescue effects (Figure S1d) similar to the observed effect in (Figure 2b). Statistically significant potentiation was observed only up to 95 min, when compared to its own baseline (*p* = 0.047, Wilcoxon) and 125 min, when compared to its control S2 (*p* = 0.040, *U* test). The baseline control potentials recorded from Figure S1(a–d) remained stable throughout the entire recording period (S2, (Wilcoxon test, *p* > 0.05, open circles)).

In general, irrespective of the type of stimulation, CSP‐TTK21 is able to rescue Aβ(1–42) induced plasticity impairments in hippocampal area CA1.

### 3.3 CSP‐TTK21 re‐establishes synaptic tagging and capture (STC) impaired by Aβ(1–42) oligomers

**FIGURE 3:**
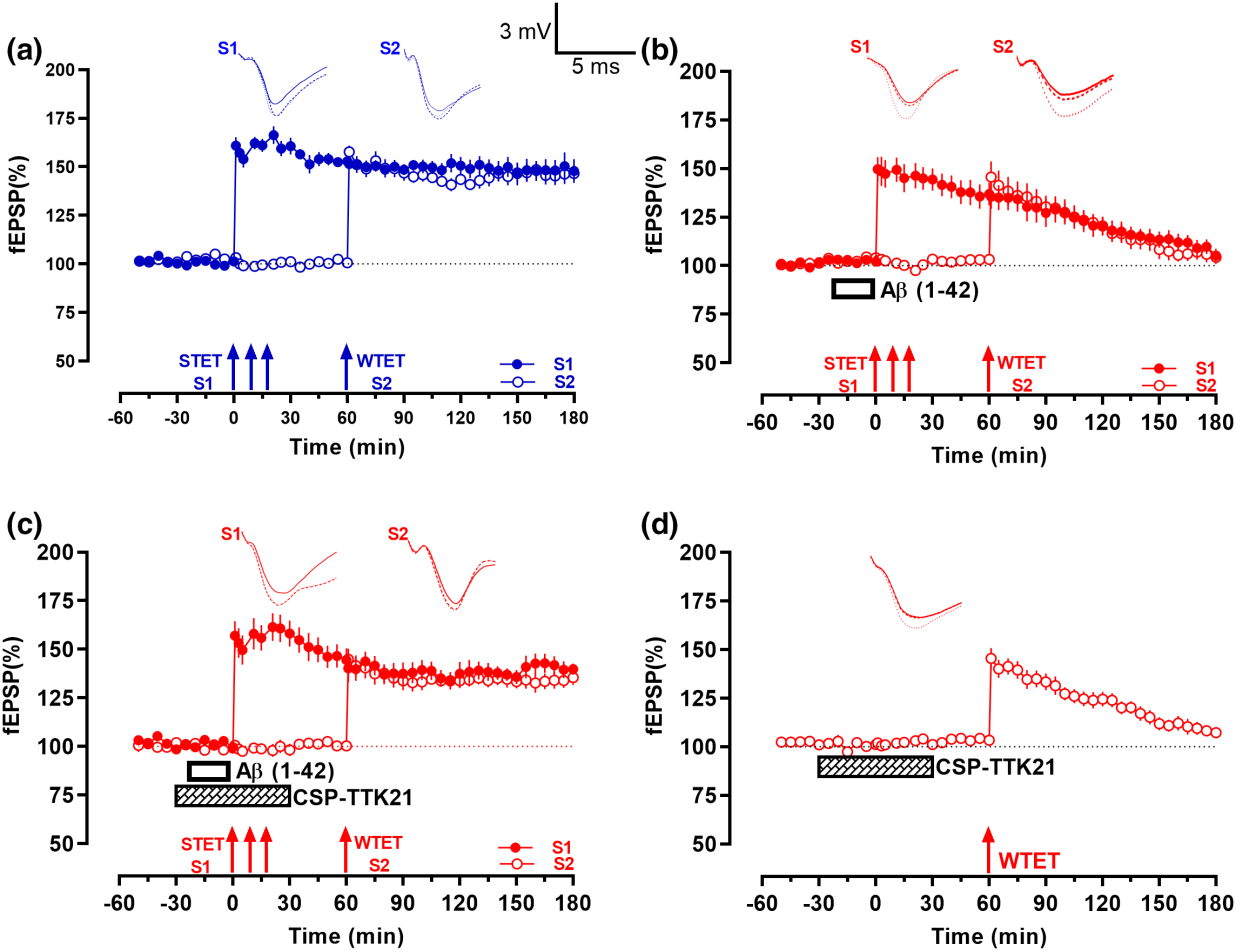
Aβ(1–42)‐impaired synaptic tagging and capture (STC) was re‐established by CSP‐TTK21. (a) Typical STC experiment in control slices. STET (solid arrows) was delivered to the first synaptic input S1 (filled circles) after a stable baseline. WTET (broken arrow) was delivered to another synaptic input S2 (open circles) 1 h after the first STET train. Potentiation in both synaptic inputs were stable throughout the recording (*n* = 7). (b) Experimental design is similar to that in (a) except that Aβ(1–42) oligomers (200 nM; open box) were bath applied 20 min before STET. Potentiation was observed in inputs S1 and S2 immediately after STET and WTET, respectively. However, fEPSP returned to baseline within 165 min and 130 min (*n* = 9). (c) Experimental design is similar to that in (b) but in addition to Aβ(1–42) oligomers application, CSP‐TTK21 (0.36 μg/ml; crisscross box) was also bath applied 30 min before and 30 min after the first train of STET. Potentiation was immediately observable after STET in S1 and after WTET in S2, respectively. The increase in fEPSP was stable for the entire recording duration (*n* = 8). (d) In this one‐pathway experiment, CSP‐TTK21 was bath applied for a total of 1 h. WTET was then delivered 30 min after the end of drug application; this resulted in a potentiation that waned over the course of the recording by 170 min (*n* = 6). All data represent mean ± SEM. Representative fEPSP traces shown in each case recorded at −30 min (solid line); 60 min (dotted line); and 180 min (hatched line). Single arrow represents the time point of application of WTET for the induction of early‐LTP. Triplet of arrows represent the point of application of STET for the induction of late‐LTP. Scale bars for all the traces, vertical: 3 mV; horizontal: 5 ms.

Synaptic associativity is a basic property of functional synaptic populations (Bin Ibrahim, Benoy, & Sajikumar, 2022; Redondo & Morris, 2011) and is impaired during aging and in AD‐like conditions (Li et al., 2017; Sharma, Dierkes, & Sajikumar, 2017; Sharma, Shetty, Arumugam, & Sajikumar, 2015). STC is one of the widely studied mechanisms of synaptic associativity at the cellular level (Bin Ibrahim, Benoy, & Sajikumar, 2022; Sajikumar, 2016). Since the CSP‐TTK21‐rescued late‐LTP is protein synthesis dependent, we tested whether the plasticity proteins from this late‐LTP can potentially be captured by nearby weakly activated synapses and thus express late‐LTP. To study STC, we used a strong before weak (SBW) protocol in which protein synthesis dependent late‐LTP was induced by STET in S1 60 min prior to early‐LTP induction in S2 by WTET (Frey & Morris, 1997; Li et al., 2017; Sajikumar et al., 2007) (Figure 3a, *n* = 7). This order of plasticity induction was employed to study STC in the CA1 area of control hippocampal slices which resulted in the expression of late‐LTP in S2 (open circles) by capturing plasticity products from the strong synaptic input S1 (filled circles). Potentiation in both S1 and S2 after tetanization stayed stable till the end of the recording period (S1: *p* = 0.016, Wilcoxon test; S2: *p* = 0.016, Wilcoxon test). In contrast to STC in control slices, Aβ(1–42) bath application prevented not only the maintenance of LTP in S1 (Figure 3b, filled circles) but also the expression of late‐LTP in S2 (Figure 3b, open circles), thus preventing both tagging and capturing processes (*n* = 8). After tetanization, fEPSP in both S1 and S2 showed an immediate increase but gradually returned to baseline. The potentiation in S1 remained statistically significant till 165 min (*p* = 0.016, Wilcoxon test) while that in S2 was statistically significant up to 130 min (*p* = 0.027, Wilcoxon test). Interestingly, bath application of CSP‐TTK21 30 min before and after the application of STET prevented the Aβ(1–42)‐induced impairment of LTP in S1 (Figure 3c, filled circles; *n* = 7), and in addition, promoted the expression of late‐LTP in S2 (Figure 3c, open circles). Both S1 and S2 displayed statistically significant potentiation at the end of the 180 min recording (S1: *p* = 0.008, Wilcox test; S2: *p* = 0.008, Wilcox test). To examine the possibility that prior application of CSP‐TTK21 itself induces metaplasticity and thus converts early‐LTP to late‐LTP in S2, we repeated the experiment shown in Figure 3(c) without STET in S1 and Aβ(1–42) application (Figure 3d). When CSP‐TTK21 was bath applied 30 min before the induction of early‐LTP, WTET resulted only in early‐LTP (Figure 3d, open circles, *n* = 6). Statistically significant potentiation was observed till 170 min after WTET (*p* = 0.031, Wilcoxon test).

### 3.4 CSP‐TTK21 leads to changes in the transcriptome and restoration of synaptic plasticity genes in the CA1 region of Aβ‐treated hippocampal slices

**FIGURE 4:**
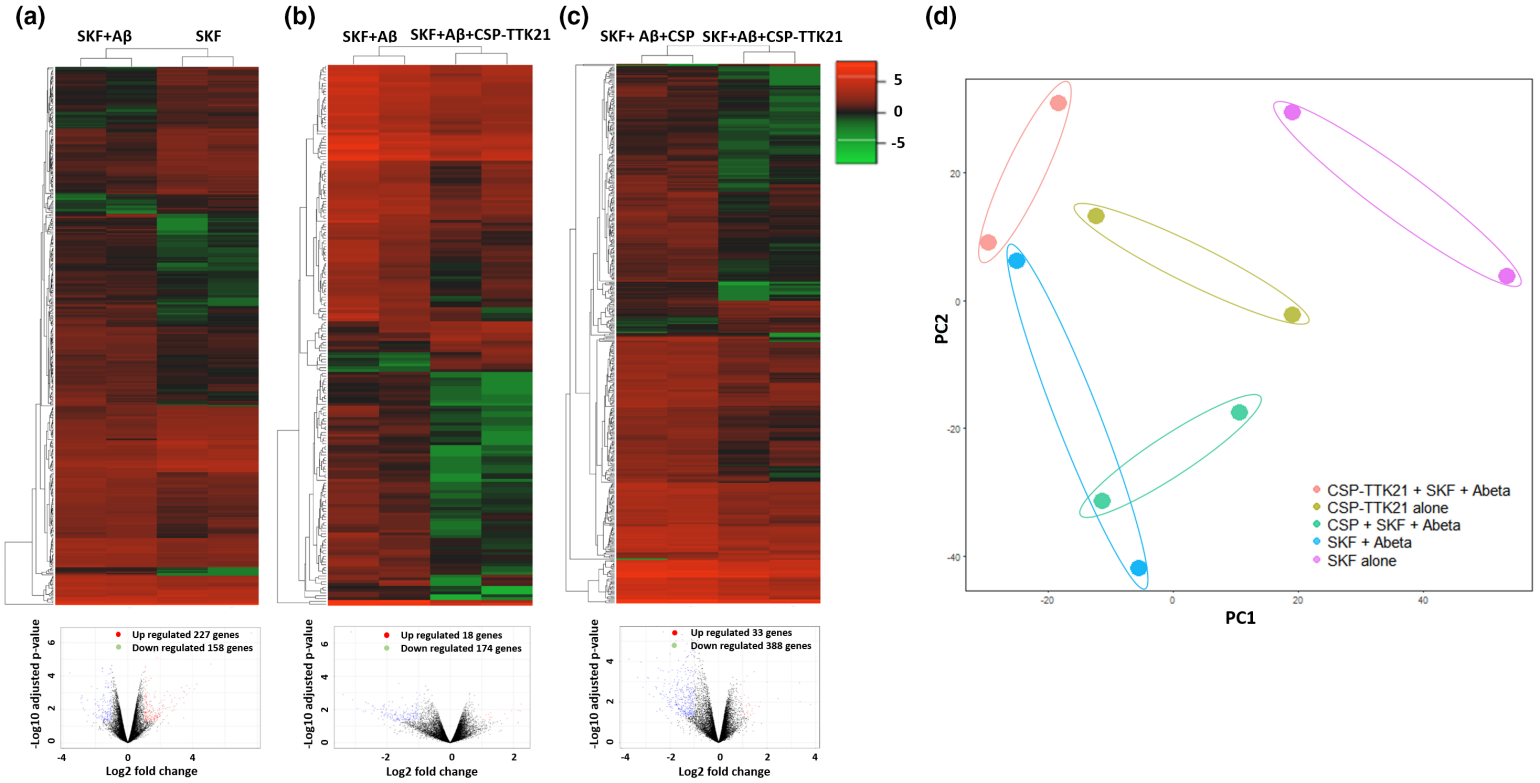
CSP‐TTK21 leads to changes in the transcriptome of the CA1 region of Aβ‐treated hippocampal slices. Unsupervised hierarchical clustering was done using Pearson uncentered algorithm with average linkage rule. Heatmaps and volcano plots representing differentially regulated transcripts in each treatment (*n* = 2, biological replicates). (a) SKF+Aβ vs SKF, (b) SKF+Aβ+CSP‐TTK21 vs SKF+Aβ and (c) SKF+Aβ+CSP‐TTK21 vs SKF+Aβ+CSP. Color coding is based on fold expression where red color indicates higher expression and green color indicates lower expression. (d) Principal component analysis of all samples used in this study, representing clusters of samples based on their similarity. Samples are color coded by treatment groups.

**FIGURE 5:**
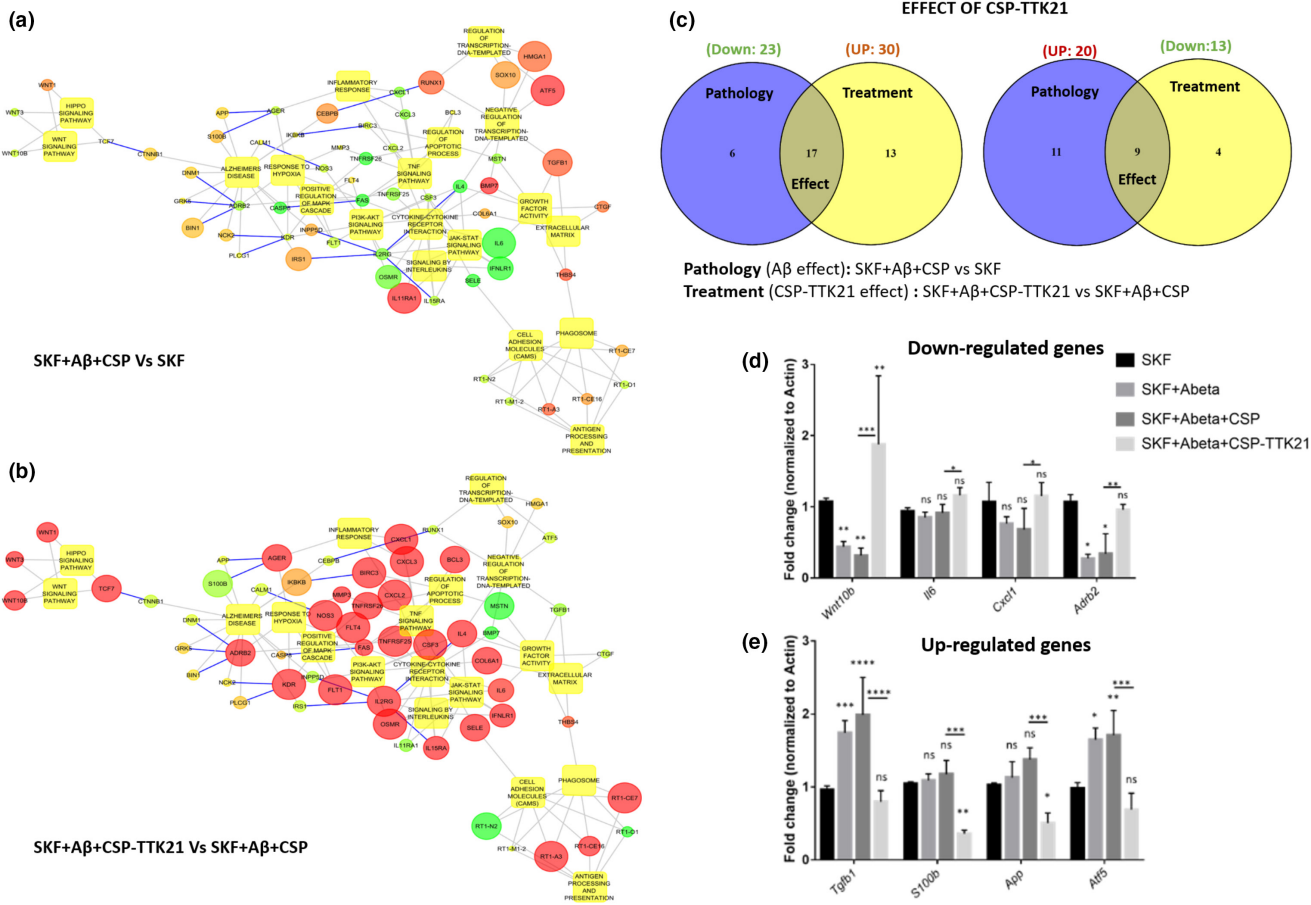
CSP‐TTK21 restores gene expression and signaling pathways impaired by Aβ(1–42) oligomers. (a) Significantly de‐regulated pathways upon SKF+Aβ+CSP co‐treatment. (b) Significantly restored pathways upon SKF+Aβ+CSP‐TTK21 treatment. The circles indicate differentially expressed genes, and boxes indicate pathways regulated by the DEGs. Up‐regulated genes are colored in red and down‐regulated are in green. Size of the circle indicates *p*‐value (the bigger the size, the lower the *p*‐value). Blue color connecting lines indicate protein–protein interaction, and gray color indicates regulation. (c) Venn diagram showing effect of CSP‐TTK21 in restoration of significantly de‐regulated genes involved in synaptic plasticity and AD progression. Blue color represents the pathological condition (SKF+ Aβ+CSP vs SKF) where 23 genes were down‐regulated and 20 genes were up‐regulated. Yellow color represents treatment condition (SKF+Aβ+CSP‐TTK21 vs SKF+ Aβ+CSP) where 30 genes were up‐regulated and 13 genes were down‐regulated. The overlap region represents genes common to both conditions and hence denotes CSP‐TTK21 treatment effect on reversal of gene expression. Expression of 17 out of 23 down‐regulated genes and 9 out of 20 up‐regulated genes in the pathological condition got reversed in CSP‐TTK21 treatment condition. (d,e) qRT‐PCR showing the expression of key genes involved in AD pathophysiology across the different treatment groups (*n* = 4, biological replicates). Two‐way anova with uncorrected Fisher's test was performed for statistical analysis, *p ˂ 0.05, **p ˂ 0.01, and ***p ˂ 0.001.

Given that the rescue of synaptic plasticity in Aβ‐treated slices by CSP‐TTK21 was dependent on de novo protein synthesis (Figure 2c,d), we hypothesized that CSP‐TTK21 might have restored functional plasticity through restoration of plasticity‐related genes expression. Electrically induced LTP using STET or TBS is input specific and the functional changes occurring during the potentiation are restricted only to the small subset of synapses, which are not enough for the gene expression studies. However, direct activation of D1/D5‐cAMP‐PKA signaling pathway by its agonist SKF 38393 can result in the global activation of synapses (not input specific) and thus can yield enough tissues for our analysis. Considering this fact, we opted for SKF‐applied tissues for gene expression studies. To investigate this, we performed RNA sequencing on the hippocampal slices from each group of rats. Slices were given 30 min of basal stimulation followed by treatment with the respective drugs and collected one‐hour post‐treatment. Upon individual comparison of each treatment group with the untreated control group, we found that SKF alone treatment resulted in 593 DEGs (Figure S2a), CSP‐TTK21 alone treatment resulted in 2141 DEGs (Figure S2b), SKF+Aβ(1–42) co‐treatment resulted in 611 DEGs (Figure S2c), SKF+Aβ(1–42)+CSP co‐treatment resulted in 823 DEGs (Figure S2d), and SKF+Aβ(1–42)+CSP‐TTK21 co‐treatment resulted in 439 DEGs (Figure S2e and Excel E1). Functional enrichment analysis revealed that most of the de‐regulated genes are involved in the regulation of gene expression, developmental processes, cell proliferation, inflammatory response, learning and memory, etc. (Figure S3 and Excel E3). Further, to delineate the molecular players involved in the re‐establishment of LTP and STC by CSP‐TTK21, we used the SKF only treated group as baseline/control to obtain the DEGs across different treatment groups (SKF+Aβ (1–42); SKF+Aβ (1–42)+CSP; SKF+Aβ(1–42)+CSP‐TTK21: and CSP‐TTK21 alone) (Figure S4). We observed that application of Aβ under SKF‐treated condition affected the expression of 385 genes of which 227 genes were up‐regulated and 158 genes were down‐regulated (Figure 4a, Excel E2). Moreover, treatment with CSP vehicle control under the SKF+Aβ treated condition when compared with the SKF+ Aβ treated group affected only 107 genes of which 83 were up‐regulated and 25 were down‐regulated (Excel E2), probably due to the glucose nature of these particles. Treatment with CSP‐TTK21 under the SKF+Aβ treated condition when compared with the SKF+Aβ treated group affected 192 genes of which 18 were significantly up‐regulated and 174 were down‐regulated (Figure 4b, Excel E2). Moreover, treatment with CSP‐TTK21 under the SKF+Aβ treated condition when compared with the SKF+Aβ+CSP treated group affected 421 genes of which 33 genes were significantly up‐regulated and 388 genes were significantly down‐regulated (Figure 4c, Excel E2). Principal component analysis (PCA) was performed to cluster the transcriptional changes for all the groups (Figure 4d). We observed that SKF+Aβ and SKF+Aβ+CSP sample clusters were close to each other representing similar pattern of gene expression and substantially different than either SKF or CSP‐TTK21 alone samples clusters correlating with altered gene expression. Collectively, these data suggest that Aβ treatment leads to transcriptional changes in these rat hippocampal slices as compared to CSP‐TTK21 and SKF alone samples. Interestingly, SKF+Aβ(1–42)+CSP‐TTK21 sample cluster was close to CSP‐TTK21 alone sample cluster and was different than SKF+Aβ and SKF+Aβ+CSP samples clusters, indicating restoration of transcriptional profile. Additionally, upon GO and pathways enrichment analysis of statistically significant differentially expressed transcripts, we found that genes involved in several pathways such as Wnt, PI3‐AKT, MAPK/ERK, and other signaling pathways implicated in the early pathophysiology of AD were significantly de‐regulated upon Aβ treatment (Figure 5a, Excel E4). A strong relationship between abnormal Wnt signaling and neuronal damaged in AD pathophysiology has been raised more than a decade ago, where it has been observed that Wnt pathway activity is impaired (Anderton et al., 2000; De Ferrari & Inestrosa, 2000). In addition, exposure of rat hippocampal neurons to Aβ results in the inhibition of canonical Wnt signaling (Alvarez et al., 2004). Moreover, several studies have shown that activation of the Wnt pathway has a neuroprotective role against Aβ peptide toxicity and improves synaptic plasticity (De Ferrari et al., 2003; Vargas et al., 2014). Interestingly, we observed an increased expression of WNT3a and WNT10B upon CSP‐TTK21 treatment (Figure 5b). Similarly, we observed a decreased ADRB2 expression in our RNA‐Seq analysis in SKF+Aβ co‐treated group (Figure 5a). This gene encodes for the β_2_‐adrenergic receptor, which activates the adenosine monophosphate/protein kinase A (cAMP/PKA) signaling cascade, leading to phosphorylation of cyclic AMP response element‐binding protein (pCREB) thus facilitating the transcription of key proteins necessary for synaptic plasticity such as brain‐derived neurotrophic factor (BDNF) (Karthivashan et al., 2019). In addition, the activation of β_2_‐adrenergic receptor has been shown to improve synaptic function and cognitive memory and has been used as a therapeutic option for various neurological disorders, such as AD, Parkinson's disease (PD), and Rett syndrome (Abdelmotilib & West, 2017; Mellios et al., 2014; Shi et al., 2018; Xu et al., 2018). Here, we observed that CSP‐TTK21 treatment increased the expression of ADRB2 gene, implicating that it plays a positive role in neuroprotection and synaptic functioning against Aβ toxicity (Figure 5b). In addition, we observed increased levels of transforming growth factor‐beta1 (TGF‐β1) signaling in the SKF+Aβ co‐treated group (Figure 5a), which has been shown to induce Aβ deposition in the cerebral blood vessels and meninges of aged mice (Wyss‐Coray et al., 1997). Increased TGF‐β1 levels have been observed in brain of AD patients (Chao et al., 1994; Peress & Perillo, 1995). TGFβ controls the expression of multiple genes associated with inflammation and immune responses. The TGFβ pathway has been associated with the regulation of genes involved in oligodendrocytes and neuronal differentiation, neuronal survival and function, and neurotransmission‐related genes (Kandasamy et al., 2014). We found that CSP‐TTK21 treatment decreased TGF‐ β1 mRNA levels, implicating that it plays a neuroprotective role against Aβ toxicity. To evaluate the direct effect of CSP‐TTK21 in synaptic plasticity and AD, DEGs associated with these processes were selected and compared across SKF+Aβ+CSP (pathology) and SKF+Aβ(1–42)+CSP‐TTK21 (effect) (Excel E5). We observed that CSP‐TTK21 rescued (increased) expression of 17 genes out of 23 genes that were down‐regulated and decreased expression of 9 genes out of 20 genes that were up‐regulated in the pathological condition (Figure 5c). These results provide direct evidence that CSP‐TTK21 restores expression of critical genes involved in synaptic plasticity and AD progression. We further validated these findings by measuring the expression of DEGs using real‐time polymerase chain reaction (RT‐PCR) (Figure 5d,e). As compared to that in SKF only treated control hippocampal slices, Aβ treatment and CSP‐Aβ co‐treatment significantly down‐regulated *Wnt10b* (Aβ: *p* = 0.009, CSP *p* = 0.001) and *Adrb2* (Aβ: *p* = 0.03, CSP: *p* = 0.02), and up‐regulated TGFβ (Aβ: *p* = 0.0005, CSP: *p* < 0.0001) and *ATF5* (Aβ: *p* = 0.01, CSP: *p* = 0.006). Interestingly, CSP‐TTK21 treatment restores the expression of *Adrb2*, *TGFβ*, and *ATF5* to the level of SKF control slices; *Wnt10b* expression was significantly increased as compared to control (*p* = 0.006).

## 4 DISCUSSION

In this study, we showed that in the hippocampal area CA1, acute exposure to exogenous Aβ(1–42) oligomers is sufficient to cause impairments in synaptic plasticity induced by distinct electrical stimuli and this impairment could be rescued by the small‐molecule CSP‐TTK21. Short‐term Aβ oligomer exposure impaired the maintenance of theta burst and high frequency induced late‐LTP and synaptic tagging and capture (STC). Not only did we show that, CSP‐TTK21 application could ameliorate the Aβ‐induced deficits in synaptic plasticity and late‐associativity, we uncovered some of the molecular changes that accompanied the rescue effect.

Aβ oligomers disrupt the ERK signaling cascade (Balleza‐Tapia & Pena, 2009), which is required for hippocampal LTP maintenance and its associated CREB‐dependent transcription (English & Sweatt, 1997; Impey et al., 1998). However, ERK signaling is not required for tag setting in late‐LTP and the induction of early‐LTP (Sajikumar et al., 2007; Winder et al., 1999). Since short‐term incubation with exogenous Aβ(1–42) oligomers has been shown to activate the ERK cascade through the α7 nicotinic acetylcholine receptor, we assume that Aβ treatment activates the ERK pathway (Dineley et al., 2001).

Several lines of evidence are linked to the dysregulation of p300/CBP in AD. It has been well established that activation of the amyloid precursor protein‐dependent signaling reduces CBP levels in primary neuronal cultures, and that Aβ peptide interferes with CREB signaling upstream of CBP (Rouaux et al., 2003; Tong et al., 2001; Vitolo et al., 2002). Additionally, diminished p300/CBP has been observed in human AD brains (Bartolotti et al., 2016; Schueller et al., 2020). Thus, we summarize that CSP‐TTK21 rescues synaptic plasticity in Aβ‐treated slices most likely by upregulating plasticity‐related proteins (PRPs) through the activation of CBP/p300 acetyltransferase activity as protein synthesis inhibitors blocked the maintenance of HFS‐LTP even in the presence of CSP‐TTK21, and that CSP‐TTK21 treatment led to a drastic increase in multiple PRPs (Chatterjee et al., 2013). However, in normal slices, CSP‐TTK21 treatment alone did not affect basal synaptic transmission neither did it reinforce early‐LTP into late‐LTP. These results imply that even when CBP/p300 is activated by CSP‐TTK21, the expression of PRPs remains tightly regulated and activity‐dependent and CSP‐TTK21 treatment alone does not affect normal neuronal function under physiological conditions (Caccamo et al., 2010). We have reported earlier that a number of PRPs play an important role in late‐LTP maintenance and STC. For instance, CaMKII, CaMKIV, and CREB are important molecules required for setting synaptic tags and PRPs while an atypical PKC isotype PKMzeta acts as an LTP‐specific PRPs. BDNF acts as a PRP for both potentiated and depressed synaptic inputs (Caccamo et al., 2010; Redondo & Morris, 2011; Sajikumar et al., 2007). Further investigations are required to confirm whether CSP‐TTK21‐induced mechanisms can activate a pool of PRPs, which allows the synapses to select the suitable PRPs and maintain long‐term plasticity. Availability of a pool of PRPs is critical for the maintenance of long‐term plasticity and associativity in aging and neurodegeneration; otherwise, the synaptic populations will eventually lose the capability to maintain synaptic plasticity through synaptic competition (Bin Ibrahim, Benoy, & Sajikumar, 2022; Sajikumar & Frey, 2004; Shivarama Shetty & Sajikumar, 2017).

High‐frequency stimulation‐induced LTP led to changes in the expression of many genes as compared to that of the untreated control. This was expected since it has long been established that synaptic changes are supported by de novo transcription and translation (Kandel, 2001; Navakkode et al., 2007). However, this plasticity‐related gene expression was disrupted by prior exogenous Aβ exposure. Our functional enrichment analysis suggests that exogenous Aβ caused the aberrant expression of genes enriched in synaptic signaling and behavioral processes, which correlates with the observed synaptic dysfunction. Other Aβ‐induced dysregulated genes were enriched in cellular development, transcription regulation, central nervous system development and neuronal genesis and differentiation. These results parallel those in gene expression profiling studies of post‐mortem tissues of AD patients (Colangelo et al., 2002; Gomez Ravetti et al., 2010).

Importantly, CSP‐TTK21 treatment partially reversed Aβ‐induced aberrant gene expression. This suggests that CSP‐TTK21, by activating the master epigenetic enzyme p300/CBP, could rescue some of the de‐regulated processes (such as synaptic plasticity and neurodegeneration) associated with AD. In particular, the CSP‐TTK21‐mediated significant restoration of Wnt, Adrb2, and Tgfβ signaling could have contributed to the functional rescue of LTP in our electrophysiology experiments, given that *Wnt* is needed for the induction and maintenance of LTP in the hippocampus and Adrb2 and Tgfβ signaling plays a crucial role in the induction and maintenance of LTP in the hippocampus (Gelinas et al., 2008; Hu et al., 2019; Mishra et al., 2019).

Furthermore, we assume that the rescue of synaptic plasticity and the enhanced expression of plasticity‐related genes might be correlated with the re‐acetylation of H2B by CSP‐TTK21, as a likely result of the acetylation of histones by p300/CBP (Chatterjee et al., 2013). Although we could not rule out the possible effects of p300/CBP activation on other non‐histone targets, our current data corroborate earlier reports that CSP‐TTK21‐mediated H2B acetylation is associated with upregulation of plasticity genes in normal and AD‐like conditions (Chatterjee et al., 2013; Chatterjee et al., 2018).

In summary, our study demonstrates that the p300/CBP activator CSP‐TTK21 can rescue Aβ‐impaired synaptic plasticity induced by various pathways, presumably through reversing Aβ‐induced dysregulation of H2B acetylation and gene expression. It further substantiates the potential use of small‐molecule activators of p300/CBP as therapeutic agents for AD.

## AUTHOR CONTRIBUTIONS

S.H.N., C.L., C.K.L.J., M.S.S., K.K.L.P, M.P.V, A.R.S, and S.N conducted electrophysiological experiments. A.K.S., S.H.S., M.V., V.J.R., I.J., and M.E. conducted biochemical experiments. A.K.S., K.K.L.P., T.K.K, S.N, and S.S. wrote the manuscript.

## CONFLICT OF INTEREST

The authors declare that they have no conflict of interest.

## Supporting information


Data S1

Figure S1

Figure S2

Figure S3

Figure S4

## Data Availability

The data that support the findings of this study are available from the corresponding author upon reasonable request. All RNAseq datasets can be uploaded from NCBI GEO: GSE206799.
