# Supplementary figures and images for: Glucose derived carbon nanosphere (CSP) conjugated TTK21, an activator of the histone acetyltransferases CBP/p300, ameliorates amyloid‐beta 1–42 induced deficits in plasticity and associativity in hippocampal CA1 pyramidal neurons
Source: Aging Cell. 2022 Aug 12;21(9):e13675. doi: 10.1111/acel.13675 (PMC9470894; doi:10.1111/acel.13675)

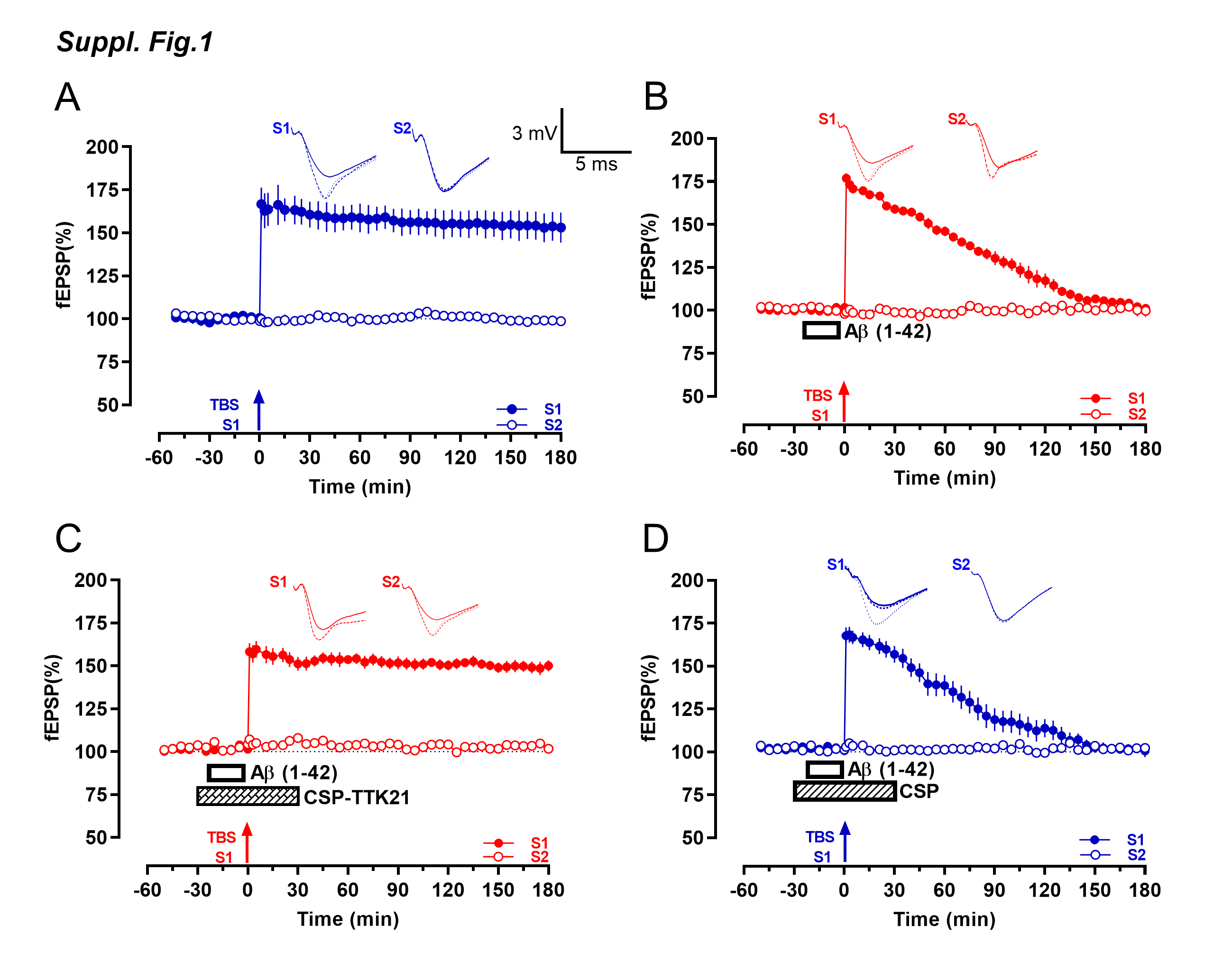

Supplement: Supplementary file 2 — Figure S1 [file ACEL-21-e13675-s004.tif]

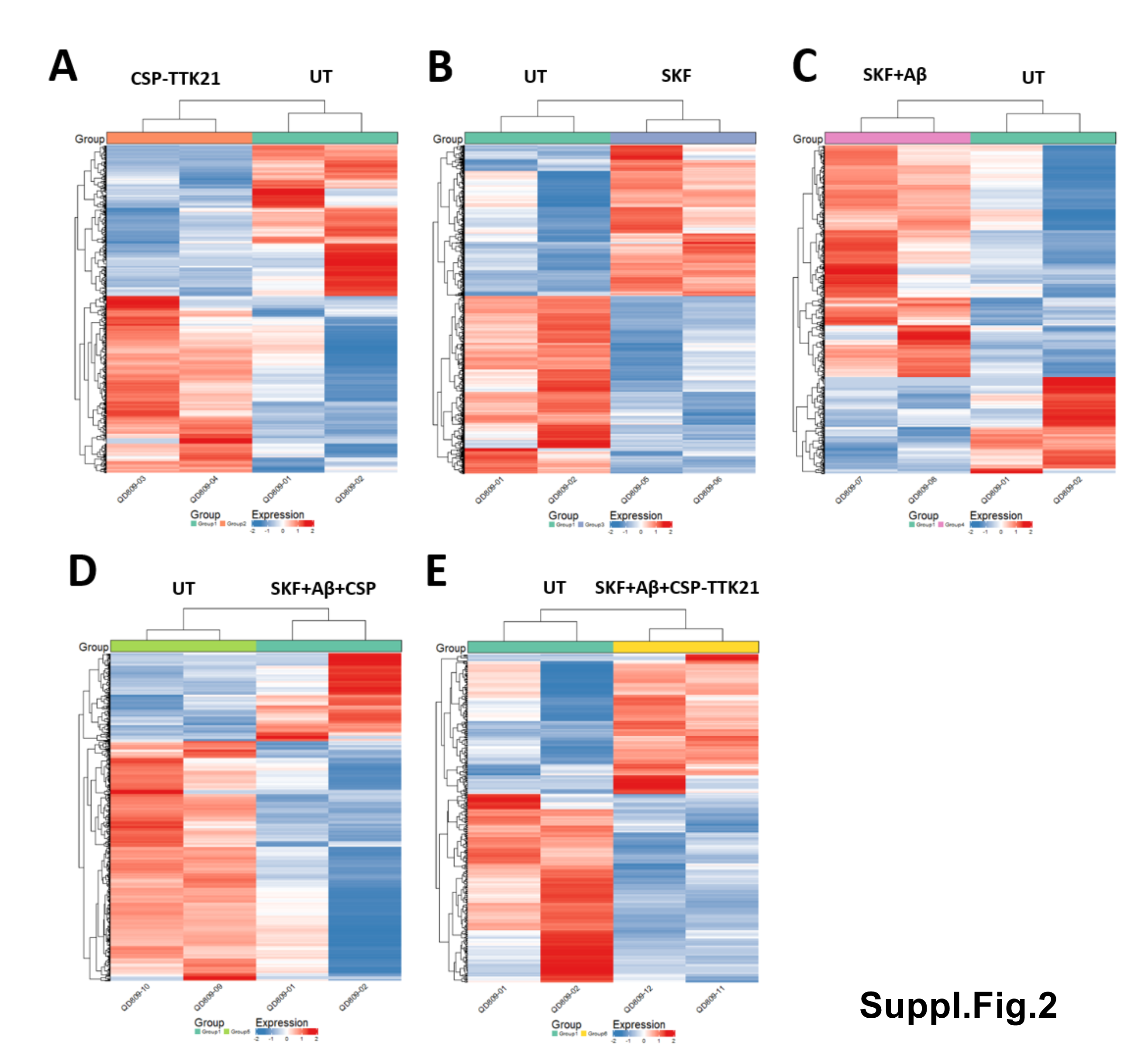

Supplement: Supplementary file 3 — Figure S2 [file ACEL-21-e13675-s002.tif]

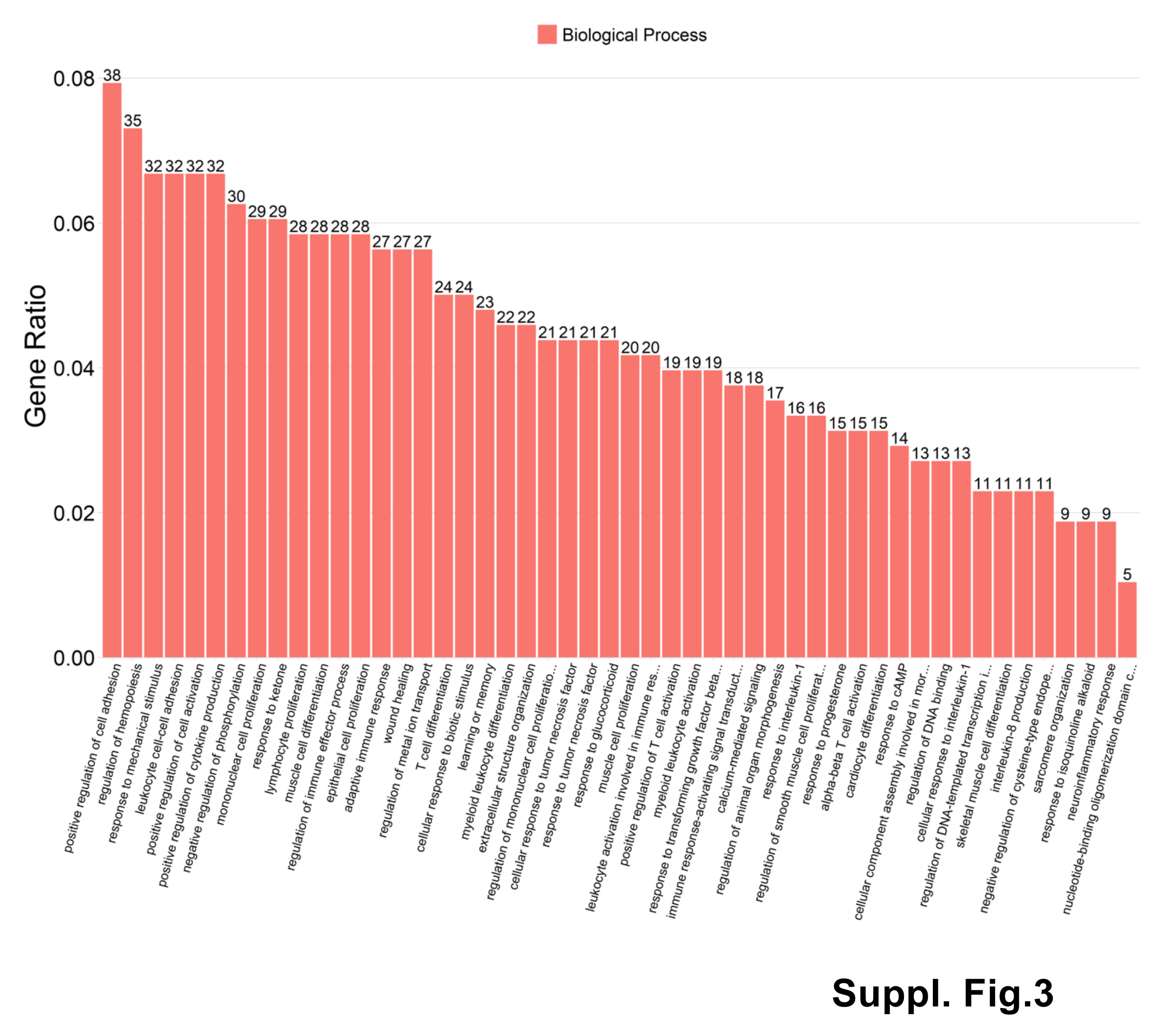

Supplement: Supplementary file 4 — Figure S3 [file ACEL-21-e13675-s005.tif]

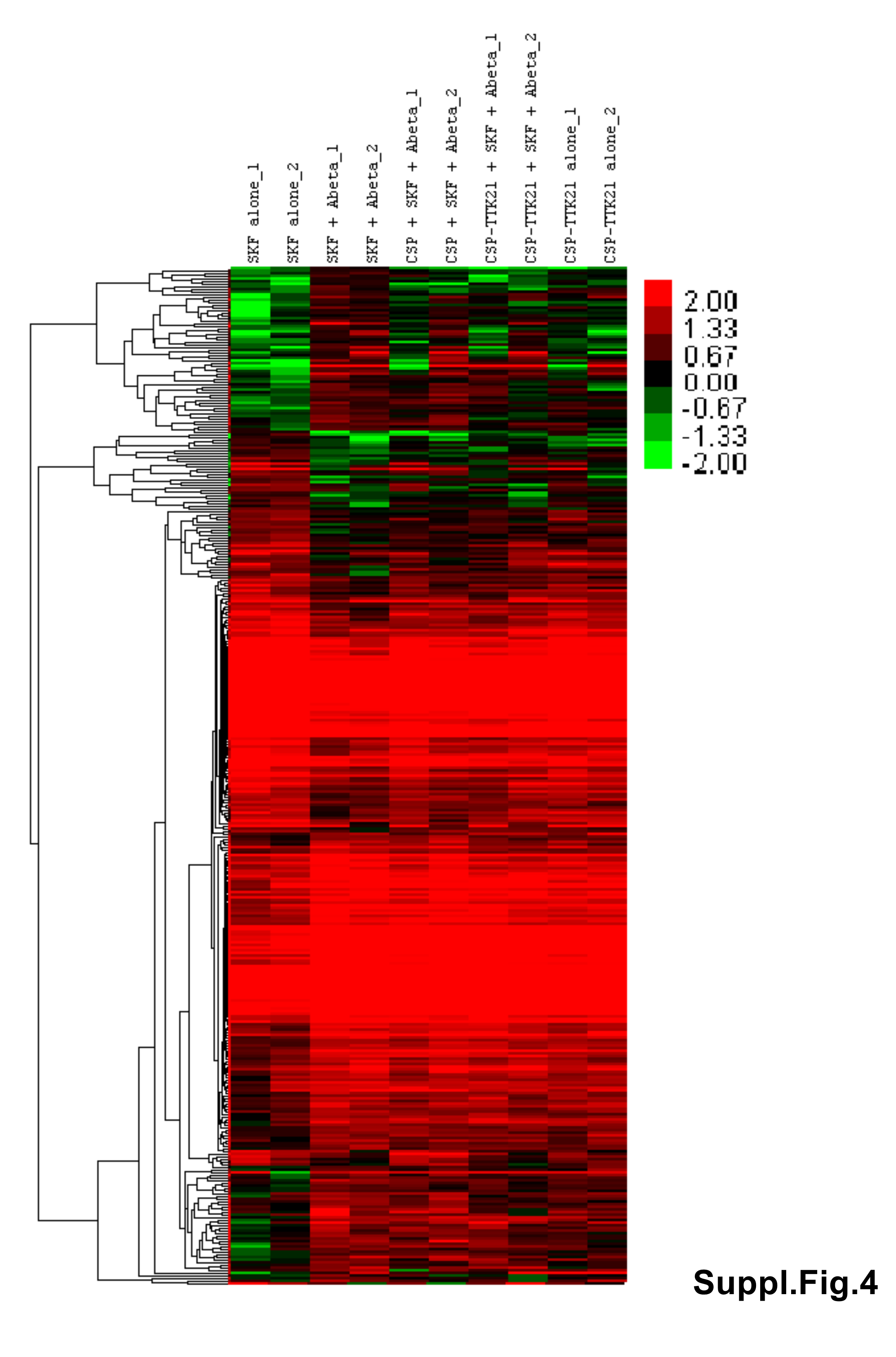

Supplement: Supplementary file 5 — Figure S4 [file ACEL-21-e13675-s001.tif]
